# An ancient *cis*‐element targeted by *Ralstonia solanacearum* TALE‐like effectors facilitates the development of a promoter trap that could confer broad‐spectrum wilt resistance

**DOI:** 10.1111/pbi.14208

**Published:** 2023-10-23

**Authors:** Niels Gallas, Xiaoxu Li, Edda von Roepenack‐Lahaye, Niklas Schandry, Yuxin Jiang, Dousheng Wu, Thomas Lahaye

**Affiliations:** ^1^ Allgemeine Genetik, Zentrum für Molekularbiologie der Pflanzen (ZMBP) Eberhard‐Karls‐Universität Tübingen Tübingen Germany; ^2^ Technology Center, China Tobacco Hunan Industrial Co., Ltd. Changsha China; ^3^ Analytik‐Zentrale Einheiten, Zentrum für Molekularbiologie der Pflanzen (ZMBP) Eberhard‐Karls‐Universität Tübingen Tübingen Germany; ^4^ Genetics, Department of Biology Ludwig‐Maximilians‐University Munich Martinsried Germany; ^5^ Hunan Key Laboratory of Plant Functional Genomics and Developmental Regulation, College of Biology Hunan University Changsha China; ^6^ Present address: Beijing Life Science Academy Beijing China

**Keywords:** *Ralstonia* injected proteins with similarity to transcription activator‐like proteins, transcription activator‐like effector, executor type resistance (*R*) gene, *Ralstonia solanacearum*, arginine decarboxylase, *Ralstonia solanacearum* species complex

## Abstract

*Ralstonia solanacearum*, a species complex of bacterial plant pathogens that causes bacterial wilt, comprises four phylotypes that evolved when a founder population was split during the continental drift ~180 million years ago. Each phylotype contains strains with RipTAL proteins structurally related to transcription activator‐like (TAL) effectors from the bacterial pathogen *Xanthomonas*. RipTALs have evolved in geographically separated phylotypes and therefore differ in sequence and potentially functionality. Earlier work has shown that phylotype I RipTAL Brg11 targets a 17‐nucleotide effector binding element (*EBE*) and transcriptionally activates the downstream *arginine decarboxylase* (*ADC*) gene. The predicted DNA binding preferences of Brg11 and RipTALs from other phylotypes are similar, suggesting that most, if not all, RipTALs target the *Brg11‐EBE* motif and activate downstream *ADC* genes. Here we show that not only phylotype I RipTAL Brg11 but also RipTALs from other phylotypes activate host genes when preceded by the *Brg11‐EBE* motif. Furthermore, we show that Brg11 and RipTALs from other phylotypes induce the same quantitative changes of ADC‐dependent plant metabolites, suggesting that most, if not all, RipTALs induce functionally equivalent changes in host cells. Finally, we report transgenic tobacco lines in which the RipTAL‐binding motif *Brg11‐EBE* mediates RipTAL‐dependent transcription of the executor‐type *resistance* (*R*) gene *Bs4C* from pepper, thereby conferring resistance to RipTAL‐delivering *R. solanacearum* strains. Our results suggest that cell death‐inducing executor‐type *R* genes, preceded by the RipTAL‐binding motif *Brg11‐EBE*, could be used to genetically engineer broad‐spectrum bacterial wilt resistance in crop plants without any apparent fitness penalty.

## Introduction

Plant pathogenic bacteria of the *Ralstonia solanacearum* species complex (*Rssc*) colonize the xylem vessels of their host plants, clogging them and causing the above‐ground parts of the plant to wilt, giving the disease its common name, bacterial wilt (Genin and Denny, 2012; Kim *et al*., 2016; Landry *et al*., 2020; Xue *et al*., 2020). Bacterial wilt is an exceptional threat to agriculture due to the global distribution of *Rssc* strains, massive crop losses associated with *Rssc* infections, broad host range of most *Rssc* strains and long persistence of *Rssc* strains in soil (Paudel *et al*., 2020). Because of its massive economic impact on global agriculture, researchers have searched for and studied genetic resistance to bacterial wilt disease in many different host species (Sharma *et al*., 2021). However, these efforts have not yet resulted in the development of a viable approach to confer broad‐spectrum resistance across all wilt‐affected crops.

We have studied *Ralstonia* injected proteins (Rips) with similarity to transcription activator‐like proteins (RipTALs), a structurally unique class of proteins present in many *Rssc* strains and, as the name suggests, homologous to transcription activator‐like effectors (TALEs) from the bacterial leaf pathogen *Xanthomonas* (de Lange *et al*., 2014). Studies on *Xanthomonas* TALE proteins, identified more than a decade before *R. solanacearum* RipTAL proteins (Bonas *et al*., 1989; Salanoubat *et al*., 2002), resulted in mechanistic insights that later studies showed also apply to RipTAL proteins. For example, both RipTALs and TALEs are injected into host cells via the type III secretion systems (T3SSs) of their associated bacterial pathogens, where they target effector binding elements (*EBEs*) in host promoters and transcriptionally activate downstream plant genes to increase host susceptibility (de Lange *et al*., 2013; Wu *et al*., 2019). Similarly, RipTAL and TALE proteins both contain a central DNA‐binding domain consisting of tandem 34/35 amino acid modules, commonly called repeats. Each RipTAL/TALE repeat pairs with a corresponding base in the *EBE* motif, with residues 12 and 13, commonly called repeat variable diresidue (RVD), determining the base preferences of each repeat (Moscou and Bogdanove, 2009; Teper *et al*., 2023). Experimental decoding of RVD base preferences, termed the RipTAL/TALE code, revealed generally identical base preferences of RVDs in the context of either RipTAL or TALE repeats. This suggests that repeats of both protein classes provide similar scaffolds for sequence‐specific interaction with DNA (de Lange *et al*., 2013).

Despite the remarkable similarities between RipTAL and TALE proteins, the two classes of proteins differ in the amino acid composition of their DNA‐binding repeats, resulting in significant differences between TALEs and RipTALs in their ability to change their DNA‐binding specificity. RipTAL repeats show comparatively low inter‐repeat similarity as their repeats not only differ in RVD but also in many non‐RVD residues. By contrast, TALE repeats differ almost exclusively in their RVDs and rarely in non‐RVD residues, and this high similarity between TALE repeats favours rearrangements within the DNA regions encoding the TALE DNA‐binding domain (Lovett, 2004). Accordingly, *Xanthomonas* TALEs are more evolvable and therefore are more likely to acquire new DNA targets over time compared to *R. solanacearum* RipTAL proteins (Nowack *et al*., 2022).

While numerous TALE plant target genes have been identified (Teper *et al*., 2023), Brg11 is currently the only RipTAL protein for which a plant target gene has been identified (Wu *et al*., 2019); therefore, at this stage, we cannot make any firm statements about the diversity of RipTAL‐targeted host genes. However, indications for RipTAL target genes can be inferred based on sequence differences between RipTALs from the four different *Rssc* lineages, known as phylotypes I‐IV (Schandry *et al*., 2016). The four phylotypes of *R. solanacearum*, are restricted to different land masses, as follows: phylotype I is found in Asia, phylotype II in the Americas, phylotype III in Africa and phylotype IV in Indonesia (Prior and Fegan, 2005). This peculiar distribution of *R. solanacearum* phylotypes suggests that continental drift, which separated the ancient supercontinent Gondwana about 180 million years ago (MYA), geographically separated a *R. solanacearum* founding population. Subsequent adaptation of strains to habitat‐specific conditions eventually led to diversification of the founding strains into four *R. solanacearum* phylotypes, which differ in genomes and traits (Wicker *et al*., 2012).

Comparison of RipTALs from the four different *R. solanacearum* phylotypes shows high similarity within a given phylotype, but low similarity between phylotypes, suggesting that phylotype‐specific sequence signatures of *RipTALs* have evolved within geographically separated *R. solanacearum* phylotypes (Schandry *et al*., 2016). Despite the low homology between RipTALs from different phylotypes, the DNA binding specificity of RipTALs from different phylotypes, inferred from their sequential RVDs using the RipTAL/TALE code, is similar for the vast majority of RipTALs (Schandry *et al*., 2016), possibly indicating that RipTALs from different phylotypes target the same host target gene. Considering that RipTALs from different phylotypes evolved from an ancestral RipTAL about 180 MYA, this may indicate that the RipTAL target gene has been maintained over a period of 180 million years.

Brg11 from the phylotype I reference strain GMI1000 is currently the only RipTAL for which an unbiased screen for plant target genes has been performed. The combined use of transcriptome profiling and *EBE* prediction in the context of tomato (*Solanum lycopersicum; Sl*) host plants revealed that arginine decarboxylase 1 (*SlADC1*) and its paralogue *SlADC2* are the only Brg11 host target genes (Wu *et al*., 2019). The 17‐nucleotide *Brg11‐EBE*, which precedes both, the *SlADC1* and *SlADC2* coding sequences (CDSs), is embedded within a ~50 nucleotide GC‐rich sequence motif known as the *ADC‐box*. The high degree of conservation of the *ADC‐box* not only in *Ralstonia* host species but also across all terrestrial plants, suggests that this sequence motif evolved around 450 MYA (Bowman, 2022). The estimated *ADC‐box* age of 450 MYA is consistent with the hypothesis that an ancient *ADC‐box*‐targeting RipTAL already existed in *Ralstonia* before the separation into four distinct *R. solanacearum* phylotypes, which occurred ~180 MYA.

In the 5′UTR of native *ADC* mRNAs, the transcribed GC‐rich *ADC‐box* is thought to hybridize into a hairpin structure. This hairpin retards ribosome movement along the *ADC* transcript, thereby reducing the translation of ADC protein. In Brg11‐dependent transcription of *ADC* genes, the RipTAL binds to the *ADC‐box* DNA motif and then hijacks the RNA polymerase II (pol II) transcription preinitiation complex (PIC) to initiate transcription ~50 nucleotides downstream of the *ADC‐box*. Since such Brg11‐induced *ADC* transcripts, unlike native *ADC* transcripts, lack the translation‐inhibiting *ADC‐box* in their 5′UTRs, they show higher translational activity than native *ADC* transcripts. Therefore, Brg11 does not simply increase *ADC* transcript levels, but induces functionally unique *ADC* mRNAs whose translation, is no longer under control of the translation‐inhibiting *ADC‐box*. Although the exact mode of action of the *ADC‐box* remains to be elucidated, it is conceivable that this ancient *cis*‐element, present in native but not Brg11‐induced *ADC* mRNAs, prevents excessive expression of ADC proteins, thereby also controlling the concentration of metabolites that depend on the enzymatic function of ADC proteins. Thus, Brg11‐induced *ADC* transcripts with their short 5′UTRs bypass the host expression control system and presumably enable the pathogen to drive ADCs to high expression levels that could probably not be achieved by translationally controlled native *ADC* mRNAs.

Arginine decarboxylase proteins catalyse the conversion of arginine to agmatine, which can be converted to the polyamine (PA) putrescine, the precursor of the PAs spermidine and spermine (Michael, 2016). Metabolite profiling revealed that Brg11‐dependent activation of *ADC* genes results in a strong increase in agmatine and putrescine, but not in spermidine or spermine (Wu *et al*., 2019). However, it is still unclear how *R. solanacearum* benefits from the Brg11‐induced changes in PA levels in host cells.

Based on our previous work, a key question that remains is whether RipTALs from other phylotypes also target the *Brg11‐EBE* within the conserved *ADC‐box* to induce transcription of host *ADC* genes. In this study, we clarified that indeed not only the phylotype I RipTAL Brg11 but also representative RipTALs from phylotype III and IV strains transcriptionally activate tomato and *Nicotiana benthamiana ADC* genes. Moreover, we revealed that activation of *ADC* genes by these RipTALs results in the same metabolic changes in host cells, further supporting our claim that Brg11 and RipTALs from other phylotypes are functionally equivalent proteins. Finally, we showed that transgenic tobacco plants containing the *Brg11‐EBE* motif upstream of the cell death‐inducing executor‐type resistance (*R*) gene *Bs4C* are resistant to RipTAL‐carrying *R. solanacearum* strains, in the absence of an apparent loss of plant fitness.

## Results

### RipTALs from different *R. solanacearum* phylotypes are predicted to target the phylotype I RipTAL *Brg11*‐*EBE* motif

**Figure 1:**
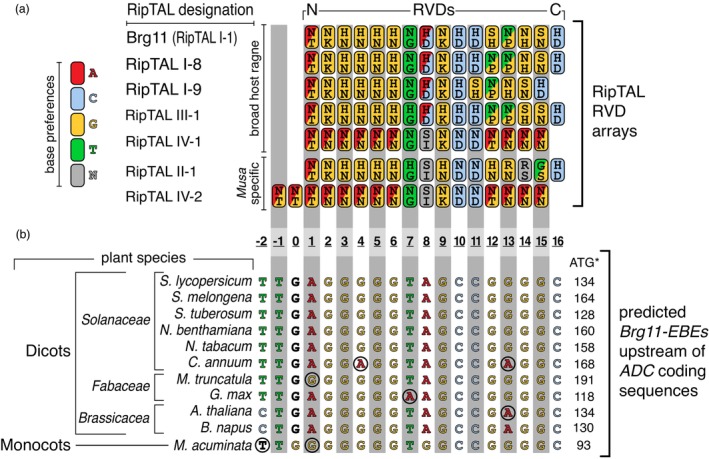
RipTALs from different *R. solanacearum* phylotypes show conservation in their predicted DNA specificities. (a) Base preferences of RipTAL RVD arrays from different *R. solanacearum* phylotypes. Repeats are shown as boxes with their RVDs (single letter code) and colours indicating nucleotide preferences. The host range of RipTAL strains is shown to the left of the repeat arrays. (b) Predicted *Brg11‐EBE* nucleotide sequences upstream of *ADC* coding sequences of different land plant species and their phylogeny (far left). Distances of predicted *Brg11‐EBEs* to the ATG start codon (ATG*) of corresponding *ADC* genes are shown on the right. Base‐colouring corresponds to repeat‐colouring shown in (a). Black font indicates predicted G0 or T0 nucleotides. Black circles highlight nucleotides that do not match with the binding preferences of aligned RVDs shown in (a). Plant species designation: *Solanum lycopersicum, Solanum melongena, Solanum tuberosum, Nicotiana benthamiana, Nicotiana tabacum, Medicago truncatula, Arabidopsis thaliana, Brassica napus, Musa acuminata*.

In previous work, we experimentally determined the DNA base preferences of different RipTAL RVDs (de Lange *et al*., 2013) and used these to deduce DNA target motifs for RVD arrays of RipTALs from different phylotypes (Schandry *et al*., 2016). To get an indication of whether the previously identified *Brg11‐EBE* motifs upstream of host *ADC* genes (Wu *et al*., 2019) would be compatible, not only with phylotype I RipTAL Brg11, but also with the DNA binding domain of RipTALs from different phylotypes, we aligned their RVD arrays to the *Brg11‐EBE* motifs upstream of *ADC* genes from different host species. Specifically, we aligned five RipTALs from broad host range *R. solanacearum* strains (Brg11, RipTAL I‐8, RipTAL I‐9, RipTAL III‐1, RipTAL IV‐1) and two RipTALs from banana‐ (*Musa*) infecting *R. solanacearum* strains (RipTAL II‐1, RipTAL IV‐2) to previously identified *Brg11‐EBEs* located upstream of either a repertoire of *R. solanacearum* dicotyledonous host species or the monocot host species, banana. Except for RipTAL IV‐2, each of the RipTALs examined aligns to the *Brg11‐EBE* in the same way as Brg11, each with no more than two RVD‐base mismatches (Figure 1). This suggests that not only the phylotype I RipTAL Brg11 but also other RipTALs from other phylotypes would bind to and transcriptionally activate *ADC* genes from a phylogenetically broad range of host species (Figure 1).

### RipTALs from broad host range *R. solanacearum* strains activate *ADC* promoters upstream of a reporter gene in an *EBE*‐dependent manner

**Figure 2:**
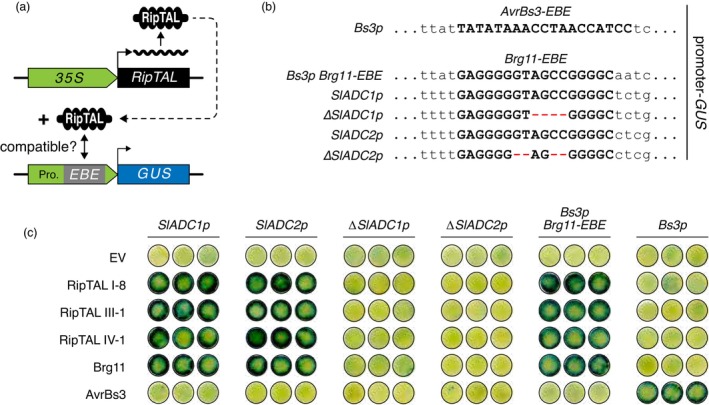
Promoter‐reporter constructs containing the *EBE* of phylotype I RipTAL Brg11 are activated by RipTALs from different *R. solanacearum* phylotypes. (a) Schematic representation of T‐DNA encoded promoter‐reporter constructs for analysis of RipTAL target specificity. *Cauliflower mosaic virus 35S* promoter‐driven (*35S*) *RipTAL* genes induce upon translation the transcription of a GUS reporter if the upstream sequence (grey box) contains a RipTAL‐compatible *EBE*. (b) Representation of GUS‐reporter upstream sequences containing Brg11‐ or AvrBs3‐compatible *EBEs* and derivatives thereof. *EBEs* are shown in bold uppercase and flanking nucleotides in regular lowercase. (c) RipTAL‐compatible *EBEs* are defined by GUS staining of *N. benthamiana* leaf discs. Staining was conducted 2 days post infiltration (dpi) of *A. tumefaciens* strains containing depicted *35S* promoter‐driven *RipTAL*/*AvrBs3* genes (left) along with strains containing depicted promoter: *GUS* constructs (top).

We first used *Agrobacterium tumefaciens*‐based promoter‐reporter assays in *N. benthamiana* leaves to experimentally investigate whether RipTALs from different broad host range *R. solanacearum* strains indeed target *SlADC1/2* upstream sequences. Specifically, we delivered T‐DNAs containing four different *35S* promoter‐driven *RipTAL* genes (*Brg11*, *RipTAL I*‐*8*, *RipTAL III‐1* and *RipTAL IV‐1*) together with T‐DNAs in which *SlADC1* or *SlADC2* upstream sequences (*SlADC1p*, *SlADC2p*) control expression of a downstream *uidA* (GUS) gene (Figure 2a). We found that the four RipTALs, which originate from three different *R. solanacearum* phylotypes, all induce both the *SlADC1p*‐ and *SlADC2p*‐driven GUS reporters. Notably, all four RipTALs failed to activate reporter constructs driven by *SlADC1p‐* or *SlADC2p*‐mutant derivatives lacking the *Brg11‐EBE* (*ΔSlADC1p*, *SlADC2p*), demonstrating that RipTAL functionality depends on the integrity of the *Brg11‐EBE* motif.

To investigate a possible context‐dependent functionality for the interaction of the *Brg11‐EBE* with different RipTALs, we studied this sequence motif in context of the pepper *Bs3* promoter (*Bs3p*), which has no sequence homology to the *SlADC1p/SlADC2p* sequences. For this, an *EBE* compatible with the *Xanthomonas euvesicatoria* (*Xeu*) TALE protein AvrBs3, which occurs naturally in the pepper *Bs3* promoter, was replaced by the *Brg11‐EBE* sequence motif. We found that all four tested RipTALs activated only the *Bs3* promoter derivative containing the *Brg11‐EBE* (*Bs3p Brg11‐EBE*), but not the native *Bs3p* (Figure 2b,c). Taken together, these data suggest that the functionality of the *Brg11‐EBE* as a target motif for different RipTALs is independent of the sequence context into which this motif is embedded.

### Sequence polymorphisms between *Brg11‐EBEs* from different host species do not affect their compatibility with RipTALs from different *R. solanacearum* phylotypes

In previous studies, we found that the 17‐nucleotide *Brg11‐EBE* is part of the *ADC‐box* motif, which is located upstream of the *ADC* CDSs (Wu *et al*., 2019). As the *ADC‐box* is highly conserved in all species of land plants, the *Brg11‐EBE* motif it contains is also highly conserved in these species. However, we found that in some species, including some *R. solanacearum* host species, the sequence differs slightly from the originally studied 17‐nucleotide *Brg11‐EBE* motif located upstream of the CDS of tomato *ADC1/2* genes. For example, the *Brg11‐EBE* upstream of the CDS of the pepper (*C. annuum*) *ADC1* gene differs by two nucleotides (Figure S1a). To investigate whether such nucleotide polymorphisms affect RipTAL‐dependent gene activation of given *ADC* genes, we transferred the *Brg11‐EBEs* from tomato, pepper, *Arabidopsis*, *Medicago* and soybean into the context of the pepper *Bs3* promoter and cloned these *Bs3* promoter derivatives upstream of the *uidA* reporter gene. *A. tumefaciens*‐mediated delivery of promoter‐reporter constructs, each containing one of five different species‐specific *Brg11‐EBE* motifs, showed that each of the four RipTALs tested activated all of the five different *Bs3* promoter derivatives (Figure S1b). In summary, our findings indicate that the sequence variations observed between *Brg11‐EBEs* from different *R. solanacearum* host plants do not affect their compatibility with any of the RipTALs tested from different *R. solanacearum* phylotypes.

### Tomato *ADC* genes are activated by RipTALs from different *R. solanacearum* phylotypes

**Figure 3:**
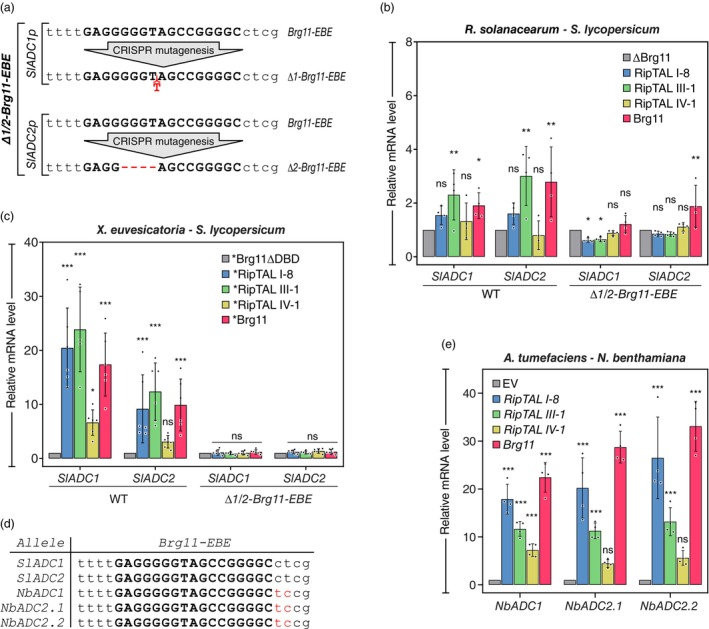
RipTALs from different *R. solanacearum* phylotypes transcriptionally activate host *ADC* endogenes. (a) CRISPR‐induced mutations in the *Brg11‐EBEs* of *SlADC1/2* genes. Red‐font dashes/letters indicate CRISPR‐induced deletions/insertions. *Brg11‐EBEs* are displayed in bold black capital letters, with the flanking residues displayed in regular lower case letters. (b) RipTAL delivery by *R. solanacearum* activates *SlADC1/2* genes in *Brg11‐EBE*‐dependent manner. Quantification of *SlADC1/2* genes by RT‐qPCR was carried out on *R. solanacearum*‐inoculated wild‐type (WT) and *Δ1/2‐Brg11‐EBE* tomatoes. Tissue from inoculated leaflets was harvested 18 h post inoculation (hpi) with *R. solanacearum* strains containing the RipTALs shown. Transcript levels are relative to the housekeeping gene *TIP41* and are normalized to expression levels of depicted tomato genotypes (WT; Δ*1/2‐Brg11‐EBE*) inoculated with *R. solanacearum* strain GMI1000, which lacks a functional RipTAL (ΔBrg11). RipTALs are delivered by the following *R. solanacearum* strains: GMI1000/phylotype I (Brg11); RUN2146/phylotype I (RipTAL I‐8), RUN369/phylotype III (RipTAL III‐1), Psi07/phylotype IV (RipTAL IV‐1). (c) Delivery of *RipTALs by *X. euvesicatoria* (*Xeu*) leads to transcriptional activation of the *SlADC1/2* genes in a *Brg11‐EBE* dependent manner. RT‐qPCR of *SlADC* genes was performed 24 hpi on tissue of *Xeu*‐inoculated WT and *Δ1/2‐Brg11‐EBE* tomato leaflets. The *Xeu* strains shown contain RipTALs with an N‐terminal type III secretion signal from the *Xanthomonas* TALE protein AvrBs3 (*RipTALs). Values are relative to the housekeeping gene *TIP41* and normalized to expression levels in tomatoes inoculated with *Xeu* containing a RipTAL lacking a DNA‐binding domain (*Brg11ΔDBD). (d) *Brg11‐EBEs* upstream of tomato and *N. benthamiana ADC* endogenes. Bold capital letters indicate *Brg11‐EBEs*, *EBE*‐flanking nucleotides are shown in regular small letters. Red font indicates nucleotide polymorphisms. (e) Delivery of RipTAL‐encoding T‐DNAs by *A. tumefaciens* leads to transcriptional activation of *N. benthamiana ADC* endogenes. RT‐qPCR of *NbADC* genes was performed on inoculated *N. benthamiana* leaf tissue, 48 hpi with *A. tumefaciens* strains containing T‐DNAs encoding depicted *35S* promoter‐driven RipTALs. Values are relative to the housekeeping gene *PP2A* and normalized to expression observed in tissue inoculated with *A. tumefaciens* containing an empty vector (EV). Asterisks denote statistical significance in comparison with control data. *P < 0.05; **P < 0.01; ***P < 0.001; ns, not significant; one‐way ANOVA with Dunnett's post hoc test versus data from control samples (ΔBrg11 [b], *Brg11ΔDBD [c] or EV [e]). Error bars represent standard deviations (SD).

Since our *Agrobacterium*‐based promoter‐reporter assays in *N. benthamiana* indicated that the RipTALs from different *R. solanacearum* phylotypes are capable of activating genes that are preceded by a *Brg11‐EBE*‐like sequence motif (Figures 2 and S1), we next quantified RipTAL‐mediated activation of *ADC* genes in the context of a more native infection scenario. Specifically, we delivered RipTALs into tomato cells via the type III secretion system (T3SS) of either the root pathogen *R. solanacearum* or the leaf pathogen *Xeu*. For quantification of transcripts by reverse transcription quantitative PCR (RT‐qPCR), we inoculated wild‐type (WT) tomato plants containing Brg11‐compatible *EBEs* upstream of the *SlADC1/2* CDSs, as well as isogenic CRISPR‐mutants (*Δ1/2‐Brg11‐EBE*; Figure 3a) lacking Brg11‐compatible *EBEs* upstream of the *SlADC1/2* CDSs (Wu *et al*., 2019). To study how RipTALs from different phylotypes affect *SlADC1/2* transcript levels, we inoculated tomato leaflets with *R. solanacearum* strains that naturally contain either Brg11, RipTAL I‐8, RipTAL III‐1 or RipTAL IV‐1. As a negative control, we included a previously established derivative of the *R. solanacearum* reference strain GMI1000 (Wu *et al*., 2019) that does not encode a functional RipTAL (ΔBrg11). RT‐qPCR‐based quantification was performed at 18 h post infection (hpi) and showed that strains carrying RipTAL III‐1 or Brg11 induced 2‐3‐fold higher *SlADC1/2* transcript levels compared to the RipTAL‐deficient *R. solanacearum* strain ΔBrg11 (Figure 3b). By contrast, RipTAL I‐8 and RipTAL IV‐1 induced in this infection assay no significant increase in *SlADC1/2* transcript levels. Notably, the RipTAL‐dependent increase in *SlADC1/2* transcripts, was strongly reduced in the tomato *Δ1/2‐Brg11‐EBE* mutant, demonstrating that the functionality of *SlADC1/2*‐activating RipTALs depends on the integrity of the *Brg11‐EBE* motif (Figure 3b). While *R. solanacearum*‐mediated delivery of some RipTALs indeed induced an *EBE*‐dependent increase in *SlADC1/2* transcript levels, the overall increase appeared small compared to the transcriptional activation of host genes typically observed upon *Xanthomonas*‐mediated delivery of TALE proteins. We reasoned that this was not due to an intrinsic functional difference between RipTAL and TALE proteins, but possibly a consequence of our experimental system. Since *R. solanacearum* is a root‐adapted xylem pathogen, our experimental assay, in which we inoculate *R. solanacearum* strains into leaf mesophyll tissue, is likely to induce immune responses that ultimately lead to RipTAL‐independent activation of *SlADC1/2* genes, which are known to be stress‐inducible (Liu *et al*., 2018; Upadhyay *et al*., 2020). These immune responses possibly interfere with *R. solanacearum*‐mediated T3SS delivery of RipTALs and consequently RipTAL‐dependent activation of *SlADC1/2* genes. To overcome these limitations, RipTALs were injected into host cells via the T3SS of the leaf pathogen *Xeu*. To achieve delivery of RipTALs via the *Xeu* T3SS, we fused a segment encoding the N‐terminal translocation domain of the *Xeu* effector AvrBs3 to each of the four studied *RipTAL* genes; these are indicated by an asterisk (*RipTAL) to distinguish them from the corresponding progenitor *RipTALs*.

Indeed, delivery of the different *RipTALs by *Xeu* generally induced higher increases in *SlADC1/2* transcripts compared to delivery of the corresponding RipTALs by *R. solanacearum* (Figure 3b,c). For example, *SlADC1* transcript levels were ~2.5‐fold higher upon infection of the *R. solanacearum* strain containing RipTAL III‐1 as compared to infection with the ΔBrg11 strain. Contrastingly, *SlADC1* transcript levels were about ~25‐fold higher upon infection with *RipTAL III‐1 by *Xeu* as compared to infection with the isogenic *Xeu* strain delivering *Brg11ΔDBD, a RipTAL protein that lacks a functional DNA binding domain. When delivered by *Xeu*, all RipTAL versus promoter combinations, except the RipTAL IV‐1 versus *SlADC2* combination, produced a statistically significant increase in *SlADC1/2* transcript levels in WT tomatoes (Figure 3c). Notably, none of the RipTAL‐delivering *Xeu* strains induced an increase in *SlADC1/2* transcript levels in the tomato *Δ1/2‐Brg11‐EBE* mutant, thereby again confirming that the functionality of all tested RipTALs depends on the integrity of the *Brg11‐EBE* (Figure 3c).

### *N. benthamiana ADC* genes are activated by RipTALs from different *R. solanacearum* phylotypes

To complement our functional analysis of RipTALs conducted in the context of tomato *ADC* genes, we extended our studies to *ADC* genes of *N. benthamiana*, which like tomato is a natural host species of *R. solanacearum*. *N. benthamiana* contains three *ADC* genes, each of which is preceded by a *Brg11‐EBE* that is sequence‐identical to the *Brg11‐EBE* motif found upstream of the tomato *ADC1/2* genes (Figure 3d). To quantify RipTAL‐dependent changes in *NbADC1/2.1/2.2* transcripts, T‐DNAs encoding RipTALs translationally fused to a C‐terminal yellow fluorescent protein (YFP) were introduced into *N. benthamiana* leaves by *Agrobacterium*‐mediated transient expression followed by RT‐qPCR. Except for RipTAL IV‐1, all RipTALs fused to YFP induced transcription of all three *NbADC* genes; RipTAL IV‐1 did so only for *NbADC1*, and it did so relatively weakly (Figure 3e).

Analysis of the YFP‐tagged RipTALs by confocal microscopy indicated that all RipTALs were expressed to similar levels (Figure S2). We, therefore, speculate that the weaker activation of *NbADC* genes by RipTAL IV‐1, which is consistent across test systems (Figure 3b–d), may be caused by the composition of its DNA binding domain, which differs in its 8th RVD from the other three RipTALs tested, possibly causing differences in the strength of its interaction with *Brg11‐EBE* (Figure 1).

Overall, our data indicate that RipTALs from different *R. solanacearum* phylotypes transcriptionally activate *ADC* genes from tomato and *N. benthamiana* in a *Brg11‐EBE*‐dependent manner.

### RipTALs from different *R. solanacearum* phylotypes induce *ADC* transcripts with short 5′UTRs

**Figure 4:**
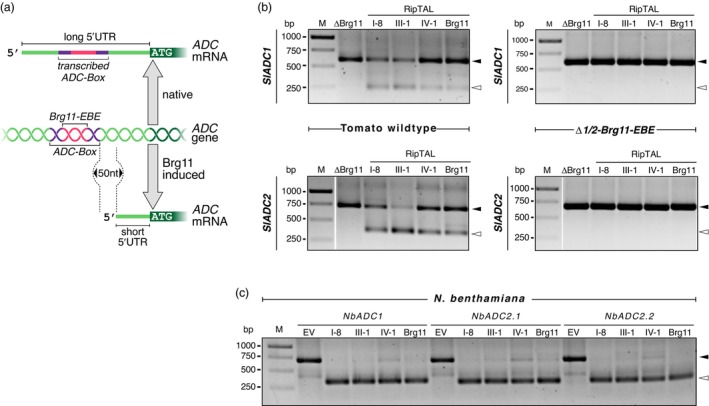
RipTALs from different *R. solanacearum* phylotypes induce *ADC* transcripts with a short 5′UTR in tomato and *N. benthamiana*. (a) Schematic representation of the native transcription of host *ADC* genes yielding mRNA with an *ADC‐box* in the 5′UTR (top) or Brg11‐induced transcription yielding a 5′‐truncated mRNA without an *ADC‐box* (bottom). (b) 5′RACE shows that 5′ truncated *SlADC1/2* mRNAs are induced by RipTALs from different *R. solanacearum* phylotypes in a *Brg11‐EBE*‐dependent fashion. 5′RACE experiments were performed with WT (left) or *Δ1/2‐Brg11‐EBE* (right) plants inoculated with *R. solanacearum* strains containing the depicted RipTALs (top). (c) 5′RACE shows that 5′ truncated *NbADC* mRNAs are transcribed in a RipTAL‐dependent manner. Depicted RipTALs were expressed in *N. benthamiana* leaves via *A. tumefaciens* mediated delivery of *35S*‐promoter‐driven T‐DNAs. White and black arrowheads in (b) and (c) indicate the long native and the short 5′ truncated *ADC* transcripts respectively. Gel pictures shown in (b) have been cropped and re‐arranged for better visualization of the differences. Non‐edited original gel pictures are shown in Figure S3.

While native *ADC* transcripts contain a transcribed *ADC‐box* in their 5′UTR, *ADC* transcripts induced by phylotype I RipTAL Brg11 have 5′‐truncated *ADC* transcripts that do not contain the *ADC‐box* (Figure 4a) (Wu *et al*., 2019). To clarify whether RipTALs from other phylotypes also induce 5′‐truncated *SlADC1/2* transcripts, we inoculated tomato leaflets with *R. solanacearum* strains containing Brg11, RipTAL I‐8, RipTAL III‐1, RipTAL IV‐1 or no functional RipTAL (ΔBrg11) and performed 5′ rapid amplification of cDNA ends (5′RACE) to identify transcription start sites (TSSs) of *SlADC1/2* transcripts. 5′RACE studies in tomato WT plants (Figure 4b, left side) showed that *SlADC1/2* transcripts with short 5′UTRs (~350 nt 5′RACE product) were detectable only upon infection with *R. solanacearum* strains containing functional RipTALs. In contrast, tissue inoculated with the ΔBrg11 strain, contained only *SlADC1/2* transcripts with a long 5′UTR (~700 nt 5′RACE product). Notably, all RipTALs failed to induce 5′‐truncated *SlADC1/2* transcripts in the tomato *Δ1/2‐Brg11‐EBE* mutant (Figure 4b, right side), demonstrating that functionality of RipTALs from different *R. solanacearum* phylotypes depends on the presence of compatible *EBEs* upstream of the *SlADC1/2* genes.

To clarify whether our findings obtained on tomato are also valid in the context of other *R. solanacearum* host species, we performed 5′RACE on RNA from *N. benthamiana* leaf tissue inoculated with *A. tumefaciens* delivering T‐DNAs (agroinfiltration) encoding *35S* promoter‐driven Brg11, RipTAL I‐8, RipTAL III‐1, RipTAL IV‐1 or no RipTAL (empty vector control). 5′RACE studies indicated that functional RipTALs induced high levels of 5′‐truncated *NbADC1/2.1/2.2* transcripts (Figure 4c; ~350 nt 5′RACE product). By contrast, *N. benthamiana* leaf tissue inoculated with *A. tumefaciens* containing the empty vector control, had exclusively *ADC* mRNAs with long 5′UTRs (~700 nt 5′RACE product).

In summary, we have shown that not only the phylotype I RipTAL Brg11 but also RipTALs from other phylotypes induce host *ADC* transcripts with short 5′UTRs in a *Brg11‐EBE*‐dependent manner, suggesting that RipTALs from different phylotypes, like Brg11, bind to and activate host *ADC* genes.

### Phylotype I RipTAL Brg11 and other *R. solanacearum* RipTALs induce similar changes in plant polyamine levels

**Figure 5:**
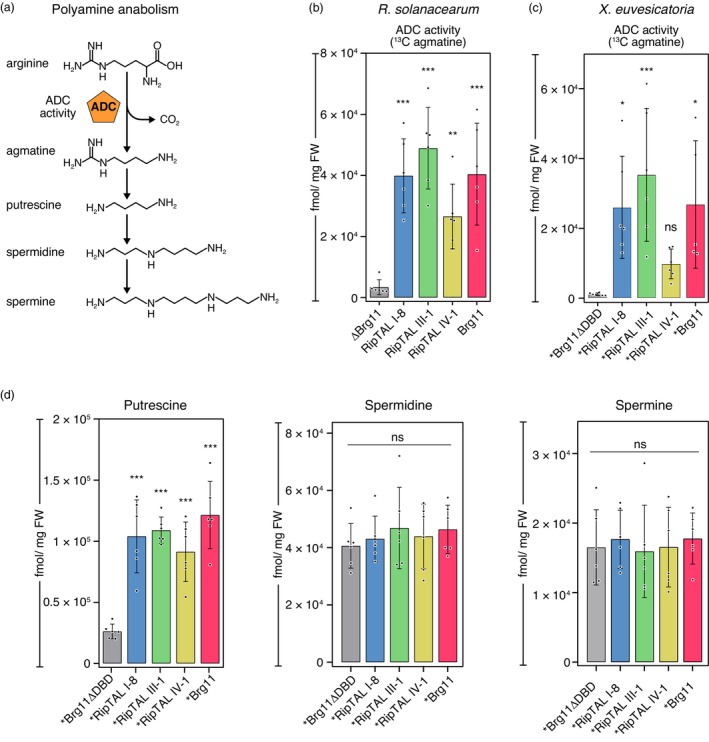
RipTALs from different *R. solanacearum* phylotypes induce similar quantitative changes in ADC‐dependent host metabolites. (a) ADC‐dependent polyamine anabolism in plants (b) RipTAL carrying *R. solanacearum* strains from different phylotypes induce increased ADC activity in tomato leaflets. Tomato leaflets were inoculated with *R. solanacearum* strains containing depicted *RipTALs* or a mutant strain lacking the *RipTAL* gene *Brg11* (*ΔBrg11*). RipTALs were delivered by the following *R. solanacearum* strains: GMI1000/phylotype I (Brg11); RUN2146/phylotype I (RipTAL I‐8), RUN369/phylotype III (RipTAL III‐1), Psi07/phylotype IV (RipTAL IV‐1). Infiltrated tissue was harvested at 18 hpi. ^13^C arginine was added to 100 mg fresh weight (FW) of homogenized plant tissue. After 3 h of incubation at 37 °C ^13^C agmatine was quantified via mass spectrometry (MS) and ADC activity was calculated. (c) *X. euvesicatoria* (*Xeu*) delivered *RipTALs from different phylotypes induce elevated ADC activity in tomato leaflets. ADC activity assays were performed as described in (b). (d) *RipTALs from different *R. solanacearum* phylotypes induce an increase in host putrescine levels but not in spermidine and spermine. Assays were carried out as described in (b). Asterisks indicate statistical significance when compared with control data. *P < 0.05; **P < 0.01; ***P < 0.001; ns, not significant; one‐way ANOVA with Dunnett's test versus ΔBrg11 (b) or *Brg11ΔDBD (d) data. Error bars represent SD.

In previous studies, we showed that the RipTAL protein Brg11 not only induces short *ADC* transcripts in tomato cells but also causes increased ADC activity, defined as the conversion of the amino acid arginine to agmatine (Figure 5a). Using a previously established mass spectrometry (MS)‐based assay that facilitates the quantification of ADC activity in tomato leaf tissue (Wu *et al*., 2019), we sought to investigate whether RipTALs from other phylotypes would also cause elevated ADC activity. Quantification of ADC activity in tomato leaflets inoculated with *R. solanacearum* strains that naturally contain Brg11, RipTAL I‐8, RipTAL III‐1 or RipTAL IV‐1 all showed significantly higher ADC activity than the ΔBrg11 control strain (Figure 5b). Similarly, inoculation of tomato leaflets with *Xeu* strains carrying *Brg11, *RipTAL I‐8, *RipTAL III‐1 or *RipTAL IV‐1 induced higher ADC activity in all cases compared to infection with the isogenic *Xeu* control strain carrying *Brg11ΔDBD (Figure 5c).

Agmatine is the metabolic product of ADC enzymes and also the precursor for ADC‐dependent biosynthesis of the polyamines (PAs) putrescine, spermidine and spermine (Figure 5a). Since our previous study showed that transcriptional activation of *ADCs* by the phylotype I RipTAL Brg11 results in increased putrescine, but not spermidine and spermine (Wu *et al*., 2019), we wondered whether RipTALs from other *R. solanacearum* phylotypes would induce similar metabolic changes. To elucidate the effect of the phylotype I RipTAL Brg11 and RipTALs from other phylotypes on plant PA levels, we inoculated tomato leaflets with *Xeu* strains carrying *Brg11, *RipTAL I‐8 *RipTAL III‐1, *RipTAL IV‐1 or *Brg11ΔDBD and quantified PA levels in infected plant tissue by MS. These studies showed that phylotype I RipTAL *Brg11 and the *RipTALs of other phylotypes only affected the concentration of putrescine, but not the concentration of the PAs spermidine and spermine (Figure 5d).

Overall, our studies show that the phylotype I RipTAL Brg11 and RipTALs from other *R. solanacearum* phylotypes induce similar changes not only at the transcriptional but also at the metabolic level.

### A RipTAL‐inducible executor transgene mediates bacterial wilt resistance in the absence of an obvious fitness penalty

**Figure 6:**
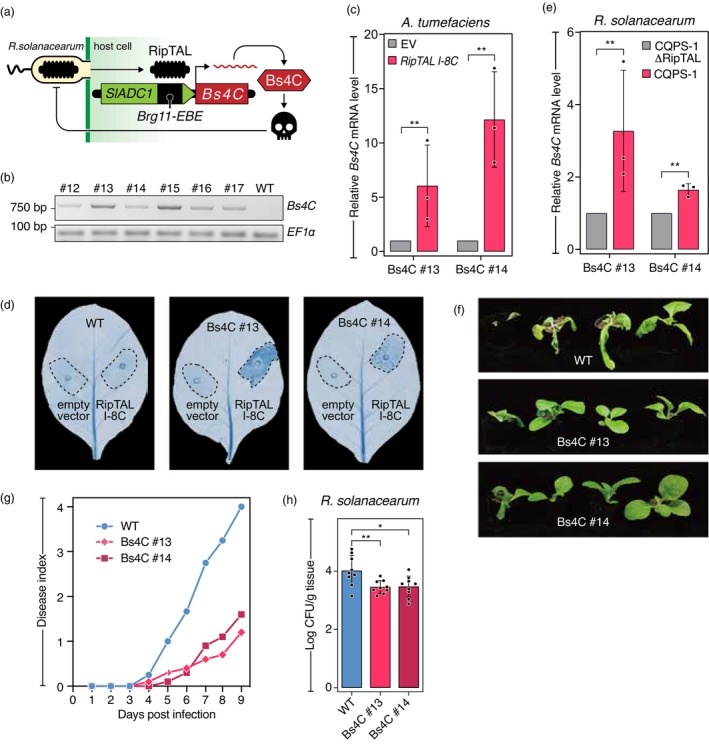
RipTAL‐dependent activation of the executor gene *Bs4C* from pepper induces wilt resistance in tobacco. (a) Schematic representation of the *RipTAL‐trap* and its function. 200 bp *SlADC1* upstream sequence (green box) containing *Brg11‐EBE* (black box), is upstream of the pepper *Bs4C* resistance gene (red square). Upon injection, RipTALs bind to the *Brg11‐EBE* and activate the cell death inducing (skull symbol) pepper *Bs4C* gene, thereby inhibiting proliferation of *R. solanacearum*. (b) PCR‐based confirmation of pepper *Bs4C* in representative transgenic tobacco lines. Amplification of the tobacco reference gene *EF1α* was used as a positive control. (c) *A. tumefaciens* mediated delivery of RipTAL I‐8C from strain CQPS‐1 activates *Bs4C* expression. *A. tumefaciens* containing a *35S* promoter‐driven *RipTAL I‐8C* T‐DNA or an empty vector (EV) control were infiltrated into leaves of the depicted *Bs4C*‐transgenic tobacco lines (Bs4C#13; Bs4C#14). *Bs4C* transcripts were quantified by RT‐qPCR, using the tobacco reference gene *EF1α* for normalization. (d) *A. tumefaciens* mediated delivery of RipTAL I‐8C triggers cell death in leaves of *RipTAL‐trap*‐transgenic tobacco lines. *A. tumefaciens* containing a *35S* promoter‐driven *RipTAL I‐8C* T‐DNA or an empty vector control were infiltrated into leaves of depicted tobacco genotypes. At 4 dpi, trypan blue staining was performed to visualize cell death. (e) *R. solanacearum*‐mediated delivery of RipTAL I‐8C activates *Bs4C* expression. *Bs4C* transcript levels were quantified by RT‐qPCR as described (c). (f) Infection phenotypes suggest that *RipTAL‐trap* containing tobacco are resistant to strain CQPS‐1. (g) Disease indexes suggests that the *RipTAL‐trap* confers resistance to *R. solanacearum*. 4‐week‐old tobacco seedlings of indicated genotypes were soil drench inoculated with *R. solanacearum*. Pictures were taken at 6 days dpi. The average disease index from 10 seedlings is shown. (h) The *RipTAL‐trap* inhibits *in planta* growth of *R. solanacearum* strain CQPS‐1. Quantification of bacteria in roots of infected plants was done at 2 dpi. Asterisks indicate statistical significance. *P < 0.05; **P < 0.01; ***P < 0.001; ns, not significant; Student's *t*‐test, comparisons as indicated in the figure.

Our finding that RipTALs from different *R. solanacearum* phylotypes all target the same ~20 nucleotide DNA sequence motif, the *Brg11‐EBE*, opens up the unique possibility of using the *Brg11‐EBE* as a RipTAL‐sensing module to control transcription of a downstream encoded cell‐death inducing plant executor *R* gene (Zhang *et al*., 2015) to confer resistance towards RipTAL‐bearing *R. solanacearum* strains. To implement this concept, we constructed a T‐DNA in which the cell death‐inducing pepper (*Capsicum pubescens*) *Bs4C* gene (Strauß *et al*., 2012) is under transcriptional control of a ~200 nucleotide sequence derived from the 5′‐upstream region of the *SlADC1* gene, containing the RipTAL‐binding *Brg11‐EBE* motif (Figure 6a). In reference to its putative function, we refer to this engineered executor *R* gene as the *RipTAL‐trap*. We transferred the *RipTAL‐trap* via *A. tumefaciens*‐mediated transformation into tobacco (*N. tabacum* cv. K326), confirmed the presence of the T‐DNA by PCR in six transgenic lines (Figure 6b), and identified two lines (#13, #14) as homozygous for the *RipTAL‐trap* in the progeny of these plants.

The *R. solanacearum* phylotype I reference strain GMI1000, commonly used to study RipTAL‐dependent host gene activation, contains two T3 effectors that induce a hypersensitive cell death reaction (HR) in *Nicotiana* species (Poueymiro *et al*., 2009) and therefore, GMI1000 was unsuitable to study RipTAL‐triggered immunity in our transgenic *N. tabacum* plants. Instead, we used the recently isolated tobacco pathogenic *R. solanacearum* strain CQPS‐1 (Liu *et al*., 2017), containing a type I‐8 RipTAL (Figure 1), to investigate the functionality of the *RipTAL‐trap* in the transgenic tobacco lines.

To clarify whether *RipTAL‐trap* transcription in the transgenic tobacco lines occurs in a RipTAL‐dependent manner, we agroinfiltrated either a T‐DNA with a *35S* promoter‐driven *RipTAL I‐8* gene from strain CQPS‐1 (*RipTAL I‐8C*) or an empty vector control into leaves of the two transgenic lines. RT‐qPCR analysis of inoculated leaf tissue revealed a 6‐ and 12‐fold increase in *Bs4C* transcript levels in transgenic lines #13 and #14, respectively, demonstrating that transcription of the *RipTAL‐trap* transgene is indeed RipTAL‐inducible (Figure 6c). Having shown that *Agrobacterium*‐mediated *in planta* expression of RipTAL I‐8 induces *Bs4C* transcription in *RipTAL‐trap* transgenic tobacco lines, we performed trypan blue staining to determine whether increased *Bs4C* transcript levels would correlate with HR. Indeed, trypan blue staining suggests that *Agrobacterium*‐mediated expression of RipTAL I‐8 triggers HR in both *RipTAL‐trap* transgenic lines but not in WT tobacco plants (Figure 6d).

To investigate whether the delivery of RipTAL I‐8C by the T3SS of *R. solanacearum* strain CQPS‐1 would lead to activation of the *RipTAL‐trap*, we generated a mutant derivative of the strain CQPS‐1 lacking a functional RipTAL, referred to as CQPS‐1ΔRipTAL. We then inoculated leaves of the two transgenic tobacco lines with either CQPS‐1 or CQPS‐1ΔRipTAL, and quantified *Bs4C* transcript levels in the inoculated tissue by RT‐qPCR. *Bs4C* transcript levels were 3‐ and 1.6‐fold higher in transgenic lines #13 and #14, respectively, when inoculated with CQPS‐1 compared to CQPS‐1ΔRipTAL (Figure 6e). Although the increase in *RipTAL‐trap* transcript levels was minor, in comparable studies, *R. solanacearum*‐mediated delivery of RipTAL Brg11 into tomato leaflets resulted in a 2‐fold increase in transcript levels of its target genes *SlADC1/2* (Wu *et al*., 2019), demonstrating that the increase in *RipTAL‐trap* transcript levels observed here is within the expected range.

Having shown that *R. solanacearum* strain CQPS‐1 transcriptionally activates the *RipTAL‐trap* transgene, we wondered whether the transgenic tobacco lines would also show enhanced resistance to *R. solanacearum* strain CQPS‐1. To analyse pathogen resistance, 4‐week‐old WT or transgenic tobacco seedlings were inoculated with the CQPS‐1 strain by soil drench and the disease index was scored visually over a period of 9 days. We found that WT plants developed more severe disease symptoms and at a much faster rate compared to the transgenic seedlings (Figure 6f,g), indicating that the *RipTAL‐trap* indeed mediates resistance to bacterial wilt disease. Similarly, quantification of *in planta* growth of strain CQPS‐1 2 days after inoculation revealed approximately 5‐fold higher bacterial counts in wild type tobacco compared to the two transgenic lines (Figure 6h), further corroborating that the transgenic lines have enhanced resistance to bacterial wilt disease.

**Figure 7:**
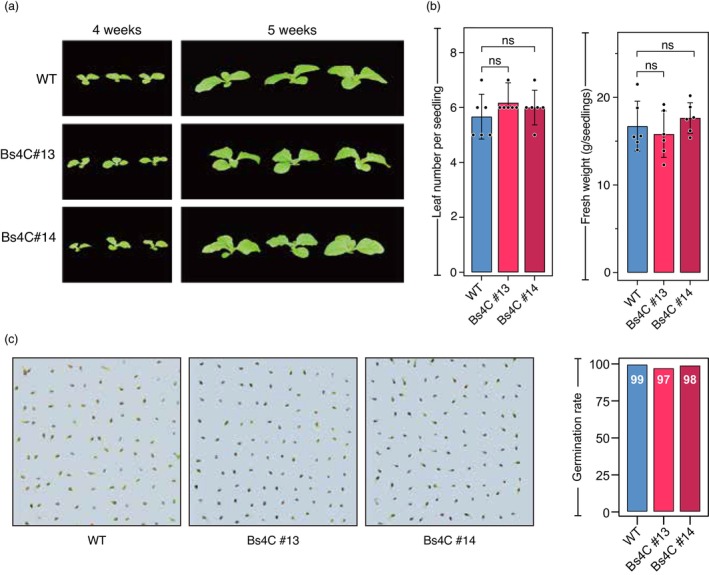
The *RipTAL‐trap* transgene has no apparent fitness penalty in the absence of pathogen infection. (a) *RipTAL‐trap* transgenic and wild‐type (WT) plants display no visible morphological differences. Plant age and genotype are shown at top and left respectively. (b) *RipTAL‐trap* transgenic and WT plants did not differ in leaf number or fresh weight. Leaf number and fresh weight was determined for 6‐week‐old plants. The bars show the mean of the analysis of six plants. Experiments were repeated three times with similar results. Asterisks indicate statistical significance. *P < 0.05; **P < 0.01; ***P < 0.001; ns, not significant; Student's *t*‐test, comparisons as indicated in the figure. (c) *RipTAL‐trap* transgenic and WT seeds show no significant differences in germination rates. Pictures on the left were taken 5 days after sowing. The quantitative analysis of the germination results is shown in the bar chart (right).

Given the known cellular toxicity of the pepper Bs4C protein encoded by the *RipTAL‐trap*, one might wonder whether leaky expression of this transgene might have a detrimental side effect on the overall fitness of the transgenic tobacco plants. However, a comparison of non‐inoculated 4‐ and 5‐week‐old WT tobacco lines with their transgenic counterparts showed no obvious differences in terms of growth and development (Figure 7a). Similarly, the average number of leaves, the fresh weight and the germination frequency did not show any significant differences between WT tobacco seedlings and the transgenic lines (Figure 7b,c), at least under the given standard, controlled conditions. Overall, the analysis of transgenic tobacco plants suggests that the *RipTAL‐trap* confers resistance to bacterial wilt disease with no apparent fitness penalty.

## Discussion

### RipTALs probably manipulate a process that was present in primeval land plants

Our work on RipTALs from different *R. solanacearum* phylotypes revealed that they all induce identical transcriptional and metabolic changes in host target cells, regardless of their phylotypic origin (Figures 2, 3, 4, 5 and S1). The functionality of RipTALs was found to strictly depend on the presence of the *Brg11‐EBE* motif in every case tested (Figures 2, 3, 4), suggesting that the binding of these RipTALs to the *Brg11‐EBE* DNA motif is the key event triggering transcription of the given downstream gene. Our results suggest that most, if not all, broad‐host range RipTALs bind to the *Brg11‐EBE* motif and induce transcription of a 5′‐truncated, translationally derepressed *ADC* mRNA. The finding that RipTALs from different phylotypes, which evolved from an ancestral RipTAL around 180 MYA, all target the same *Brg11‐EBE* motif suggests that the ancestral RipTAL already targeted this motif, and that this targeting specificity has been maintained in RipTALs from different phylotypes over 180 million years of RipTAL evolution. Since the RipTAL target sequence is present in all land plants (Wu *et al*., 2019) and has been maintained as a target for over 180 million years of evolution, this suggests that RipTALs manipulate a process that was already established in early land plants and has not changed significantly for 180 million years.

### Similarities between RipTALs and TALEs can be used to infer the minimum age of an ancestral precursor protein

RipTAL and TALE proteins both bind target DNA with tandem arranged modules, each binding one base in targeted DNA sequence. The correlation between specific RVDs and their DNA base preferences, known as the RipTAL/TALE code, is largely identical in RipTAL and TALE proteins, suggesting that both classes of proteins form very similar scaffolds to interact with host DNA in a sequence‐specific manner (de Lange *et al*., 2013). This extraordinary similarity in the structure–function relationship of the DNA‐binding domains of RipTAL and TALE proteins argues in favour of common ancestry.

A unique feature of TALE‐induced host transcripts is that their TSS is generally ~50 nucleotides downstream of the respective TALE *EBE* and is often different from the TSS of the corresponding native transcripts (Antony *et al*., 2010; Hummel *et al*., 2012; Römer *et al*., 2009a,b; Strauß *et al*., 2012; Tian *et al*., 2014; Tran *et al*., 2018; Wang *et al*., 2015). Similarly, previous work on the phylotype I RipTAL Brg11 showed that the TSSs of Brg11‐induced *ADC* transcripts are generally ~50 nucleotides downstream of the *Brg11‐EBE*. (Wu *et al*., 2019). In our current work, 5′RACE studies have revealed that this spatial relationship between the *Brg11‐EBE* and the TSS of the induced mRNAs is observed not only for transcripts induced by the phylotype I RipTAL Brg11, but also for transcripts induced by RipTALs from other phylotypes (Figure 4). The ~50 nucleotide distance between the *EBEs* and the TSS of induced mRNAs is therefore a common structural feature observed in RipTAL‐ and TALE‐induced host transcripts. This observation provides further support for the hypothesis that the RipTAL and TALE proteins evolved from a common ancestor prior to the divergence of a founder population of *R. solanacearum* into four phylotypes at around 180 MYA.

### Functional features of executor *R* genes enable pathogen resistance without yield penalty

Molecular analysis of TALE‐dependent immune responses in monocot and dicot host plants led to the identification of executor‐type *R* genes that, induce host cell death upon expression, thereby preventing the spread of the biotrophic bacterium *Xanthomonas* (Kraepiel and Barny, 2016). TALE‐dependent transcription of executor *R* genes is relies on upstream *EBEs*, which tether TALEs to DNA and exploit their virulence activity to activate transcription of downstream encoded executor proteins.

Our findings demonstrate that RipTAL proteins of different *R. solanacearum* phylotypes all target the 17‐nucleotide *Brg11‐EBE* motif, which is part of the *ADC‐box*, a 50‐nucleotide *cis*‐element located upstream of land plant *ADC* genes. We therefore speculated that an executor *R* gene preceded by the *Brg11‐EBE* motif would mediate resistance against RipTAL‐containing *R. solanacearum* strains. To test this hypothesis, we placed ~200 nucleotides of the tomato *ADC1* upstream sequence, containing the *Brg11‐EBE* motif, upstream of the executor gene *Bs4C* from pepper (Figure 6a). *Ralstonia* infection studies of transgenic tobacco lines containing this T‐DNA construct, termed the *RipTAL‐trap*, indeed showed that executor gene transcription was RipTAL dependent and that bacterial growth and symptom development were significantly reduced in the transgenic lines (Figures 6 and 7).

An obvious concern associated with using executor genes is that leaky expression may have unwanted side effects. In this context, it is noteworthy that the ~200 nucleotide sequence containing the RipTAL‐binding *Brg11‐EBE* motif originates from the 5′UTR, not from the tomato *ADC1* promoter. Although the *SlADC1* 5′UTR is unlikely to have intrinsic promoter activity, leaky transcription of the *RipTAL‐trap* may occur due to the integration of the T‐DNA near active promoter regions of the tobacco genome. This type of leaky transcription would result in mRNAs where the translation‐inhibiting *ADC‐box* precedes the *Bs4C* CDS, potentially reducing Bs4C expression to levels too low to trigger HR. If, in a transgenic line, the specific integration site of the transgene in the genome causes not only leaky transcription but also translation of the executor gene, this is likely to cause developmental defects already at the callus stage, allowing such undesirable plants to be identified and eliminated at an early stage.

An important aspect in the development of transgene‐based, pathogen‐resistant crops is that the respective transgene should have no adverse effect on plant performance. Typically, however, resistance‐conferring transgenic systems are based on one or more constitutively expressed components, such as an effector‐specific receptor protein (Vuong *et al*., 2022). This not only places an energetic burden on the plant but also carries the risk that constitutive expression will become the target of host gene silencing, ultimately resulting in a lack of receptor expression and a dysfunctional perception system (Rajeevkumar *et al*., 2015).

As RipTAL/TALE‐recognizing promoter traps are not protein‐ but DNA‐based receptors, their functionality does not depend on a constitutively expressed functional components and therefore, do not represent an energetic burden for the plant in the absence of pathogen attack. Furthermore, without a constitutively expressed component, such *RipTAL/TALE‐trap* transgenes are unlikely to be inactivated by host gene silencing. In summary, these considerations, along with the data presented, suggest that *RipTAL/TALE‐trap* transgenes can confer pathogen resistance without yield penalty.

### The *RipTAL‐trap* is likely more durable than executor *R* genes that mediate recognition of *Xanthomonas* TALE proteins

A key question for the agricultural application of the *RipTAL‐trap* is whether it confers durable resistance to *R. solanacearum*. We envision two scenarios for overriding the *RipTAL‐trap*: 1. *R. solanacearum* strains evolve that lack functional RipTALs, or 2. *R. solanacearum* strains evolve RipTALs that activate *ADC* genes by binding outside the *ADC‐box* motif. The first scenario seems plausible as strains lacking functional RipTALs have already been collected in natural habitats (Schandry *et al*., 2016). Whether RipTAL‐lacking strains are less virulent than RipTAL‐containing strains in an agricultural setting remains unclear. However, our observation that *ADC*‐activating RipTALs have been maintained for >180 million years suggests that RipTALs promote virulence and that *R. solanacearum* populations without RipTAL‐containing strains have reduced virulence in native infection scenarios. The second scenario, in which *R. solanacearum* evolves RipTALs with altered DNA binding specificity targeting *ADC* upstream sequences distinct from the *ADC‐box* region integrated into the *RipTAL‐trap*, seems plausible at first sight and is supported by recent small‐scale laboratory studies. These studies have shown that within ~1000 bacterial generations, xanthomonads can adapt the sequence specificity of TALE DNA binding domains to activate virulence‐enhancing host genes (Teper and Wang, 2021). Yet, while this scenario seems likely for TALE‐specific executor *R* genes, the unique features of the RipTAL plant target sequence suggest that the *RipTAL‐trap* will be more durable. The RipTAL target, the *ADC‐box*, is not a random *ADC* upstream sequence selected by RipTALs as an *EBE* to induce *ADC* transcripts, but it serves two other purposes: (1) RipTALs have probably evolved to target this *cis*‐element since the *ADC‐box* is conserved in all host plants, enabling RipTALs to bind to and activate *ADC* genes across all host plants. (2) By binding to the *ADC‐box* DNA motif and inducing transcripts ~50 nucleotides downstream of this *EBE*, RipTALs induce a unique type of *ADC* transcript that, unlike native *ADC* transcripts, lacks the translation‐inhibiting *ADC‐box* in its 5′UTR, resulting in high levels of ADC expression. Because of these two distinct, potentially virulence‐promoting properties associated with the use of the *ADC‐box* as an *EBE*, it seems unlikely that RipTALs will rapidly evolve to target areas outside the *ADC‐box* to activate *ADC* genes. In summary, several lines of evidence suggest that our *RipTAL‐trap* will provide more durable resistance than native TALE‐specific executor‐type *R* genes.

### Implementation of the *RipTAL‐trap* concept in non‐transgenic crop plants

Here we present a concept for engineering wilt‐resistant plants based on the coupling of the RipTAL sensor motif *Brg11‐EBE* with the cell death‐inducing executor transgene *Bs4C* from pepper (Figure 6a). Genes encoding Bs4C‐like proteins are present in all Solanaceae genomes (Strauß *et al*., 2012). Therefore, cell death‐inducing *Bs4C* endogenes could functionally replace the pepper  *Bs4C* transgene if they were preceded by the *Brg11‐EBE* motif. A groundbreaking study in rice has recently shown that oligonucleotide‐based targeted insertion facilitates the placement of any desired *EBE* upstream of a selected executor endogene (Kumar *et al*., 2023). Thus, the molecular tools are available to place the *Brg11‐EBE* upstream of a cell death‐inducing *Bs4C* endogen, which would presumably result in crops resistant to RipTAL‐carrying *R. solanacearum* strains. Since our approach of using gene editing to insert RipTAL binding motifs upstream of executor endogenes is classified for regulatory purposes as a non‐transgenic approach in most countries of the world (Buchholzer and Frommer, 2023), it should make it possible to breed and deploy non‐transgenic bacterial wilt‐resistant plants in the future.

## Experimental procedures

### Bacterial strains and growth conditions

*Agrobacterium tumefaciens* GV3101 was transformed with pGWB614 or pGWB3* (Nakagawa *et al*., 2007) containing *RipTAL*‐overexpression or *uidA*‐reporter constructs and cultured in YEB medium with rifampicin and appropriate antibiotics at 28 °C. *R. solanacearum* CQPS‐1 was kindly provided by Wei Ding and Ying Liu (Southwest University). CQPS‐1ΔRipTAL was generated and validated as previously described (Wu *et al*., 2019). Primers used are listed in Table S1. *R. solanacearum* strains used are listed in Table S2. *R. solanacearum* were cultured in B medium (1% peptone, 0.1% tryptone, 0.1% yeast extract, 2.5% glucose) without or with gentamycin at 28 °C. *X. euvesicatoria* (*Xeu*) 85‐10 was transformed with pDSK602:*RipTALs to generate *RipTAL‐delivering derivatives. *Xeu* were cultured in NYG (0.5% peptone, 0.3% yeast extract, 0.2% glycerol) supplemented with spectinomycin and rifampicin at 28 °C.

### Plant material

Tomato and *N. benthamiana* were grown in a growth chamber at 21 ± 1 °C with a 16‐h light and 8‐h dark photoperiod and a relative humidity of 30%–50%.

### Generation of expression constructs

Level I modules were generated by PCR and cloned into pUC57 as described (Binder *et al*., 2014). *RipTALs* and promoters were cloned into pENTR using Golden Gate cloning and transferred into pGWB641 or pGWB3* by Gateway cloning. *RipTALs* were cloned downstream of the AvrBs3 T3SS into pENTR to generate *RipTALs that were then cloned into pDSK602 via BamHI and HindIII for expression in *X. euvesicatoria*.

### Generation of *SlADC1p*‐driven *Bs4C* transgenic tobacco plants

*SlADC1p* (173 bp) was amplified from tomato gDNA. *Bs4C* was synthesized by Sangon Biotech. Both were assembled into pCHF3 and transformed into *N. tabacum* K326 using *A. tumefaciens* LBA4404 as previously described (Li *et al*., 2022). T0 plants were screened on MS medium containing 50 mg/L kanamycin and genotyped with transgene‐specific primers.

### *uidA* reporter assays in *N. benthamiana*

*Agrobacterium tumefaciens* carrying *RipTAL* overexpression or reporter constructs were harvested from overnight cultures by centrifugation and resuspended in infiltration buffer (pH 5.6, 10 mM MgCl_2_, 5 mM MES and 150 mM acetosyringone) to OD_600_ = 0.8. Overexpression and reporter strains were mixed in equal amounts to a final OD_600_ = 0.4 and infiltrated into leaves of 4‐week‐old *N. benthamiana* plants with a blunt‐end syringe. GUS assays were performed as described (Strauß *et al*., 2012).

### *R. solanacearum* and *Xeu* assays in tomato

Four‐ to five‐week‐old tomato plants were transferred to a 28 °C (80% rel. humidity) 16‐h light, 26 °C (70% rel. humidity) 8‐h dark cycle 24 h before *R. solanacearum* infection. The next day, tomato leaflets were infiltrated with *R. solanacearum* strains GMI1000, RUN2146, RUN369, Psi07 or ∆*Brg11* mutant strain at OD_600_ = 0.1 in water using a blunt‐end syringe. For assays with *Xeu* carrying pDSK602:*RipTALs, leaflets of 4‐ to 5‐week‐old tomatoes were infiltrated at OD_600_ = 0.4 in 1 mM MgCl_2_ using a blunt‐end syringe. Tissues for RNA extraction were harvested at 18 hpi for *R. solanacearum* and at 24 hpi for *Xeu*.

### *R. solanacearum* infection assays in *N. tabacum* (tobacco)

Four‐week‐old tobacco plants, grown in individual pots, were inoculated with *R. solanacearum* by pouring 15 mL of OD_600_ = 0.1 bacterial suspension. Disease index was recorded using a scale from 0 to 4 (0: no wilted leaves; 1: 1%–25% wilted leaves; 2: 26%–50% wilted leaves; 3: 51%–75% wilted leaves; 4: 76%–100% wilted leaves). For *in planta* bacterial growth studies, root tissue nearby the stem base was cut, surface sterilized, weighted and ground in 1 mL of sterile water. Dilutions were plated on agar and incubated at 28 °C for 36–48 h. For *R. solanacearum* infiltration, bacterial suspensions were diluted to 10^4^ cfu/mL and infiltrated into leaves with a blunt syringe. For transient expression, *A. tumefaciens* GV3101 containing binary constructs were harvested by centrifugation, resuspended in sterile water adjusted to OD_600_ = 0.4 and infiltrated into leaves of 4‐ to 5‐week‐old tobacco seedlings with a blunt‐end syringe.

### RT‐qPCR

Plant RNA was extracted using the Universal RNA Purification Kit from EurX (E3598). Five hundred nanograms of RNA was transcribed into cDNA using the RevertAid cDNA Synthesis Kit (K1622). RT‐qPCR was performed as previously described (Wu *et al*., 2019). Each sample was analysed in three technical replicates. PCR was performed using a CFX96 system (Bio‐Rad) *NtEF1α* was used as a reference gene for normalization. Relative expression of target genes was calculated using 2^−ΔΔCT^ and divided by the respective negative control to obtain fold‐changes.

### 5′ rapid amplification of cDNA ends (5′RACE)

For 5′RACE, the SMARTer RACE 5′/3′ kit from Clontech/Takara was used. RNA samples were synthesized into adapter‐ligated cDNA by the SMARTScribe reverse transcriptase. Nested PCR was performed to amplify *ADC* transcript variants.

### ADC activity assay and polyamine analysis

Frozen, minced plant tissue (50 mg) was homogenized in ice‐cold 300 μL ADC activity buffer (5 mM Tris pH 8.5, 1 mM ascorbate, 50 μM pyridoxal 5 phosphate, 0.75% polyvinylpolypyrrolidone (PVPP), protease inhibitor solution (1 tablet Roche cOmplete™ in 5 mL buffer), 50 μM U‐^13^C_6_ L‐arginine (Eurisotop, Germany) and 50 μM L‐tryptophane‐D5 as internal standard (Sigma‐Aldrich) and incubated at 37 °C (3 h). The reaction was stopped with 20 μL heptafluorobutyric acid (HFBA). After centrifugation (18 600 g, 30 min, 4 °C), the supernatant was subjected to metabolite analysis. Five to six biological replicates were measured per genotype. LC–MS profiling was performed as reported previously (Wu *et al*., 2019) using a Micro‐LC M5 and a QTRAP6500+ (Sciex) operated in MRM mode (see Table S3 for settings). Transitions monitored for each metabolite are displayed in Table S4.

### Germination assay

Tobacco seeds were surface sterilized with 75% ethanol for 2 min and 10% NaClO (v/v) for 2 min, followed by three washes with sterile water. Sterilized seeds were then placed on a filter paper pre‐moistened with sterile water, transferred to Petri dishes and incubated at 24 °C. The germination rate was monitored and recorded 5 days after incubation.

### Trypan blue staining

*Agrobacterium tumefaciens* containing an empty vector or *35S* promoter‐driven *RipTAL I‐8C* were infiltrated into 4‐week‐old tobacco leaves. Trypan blue staining was done 4 days post‐infiltration. Trypan blue stock solution was prepared by mixing the following components: phenol 10 g, glycerol 10 mL, lactic acid 10 mL, water 10 mL and trypan blue 0.02 g. The stock solution was then diluted with 96% ethanol (1:2, v/v) to create the working solution. Infiltrated leaves were immersed in the trypan staining work solution and boiled for 1 min, followed by incubation in the working solution for an additional 24 h. Afterwards, the stained leaves were incubated in a chloral hydrate solution (2.5 g of chloral hydrate dissolved in 1 mL of water) for 72 h. Finally, the destained leaves were scanned.

## Author contributions

N.G. carried out transcript studies on *ADC* endogens. N.G. and E.R. carried out metabolite profiling. N.S. cloned *RipTAL* genes from *R. solanacearum* strains. D.W., Y.J. and X.L. generated and studied tobacco transgenic lines. T.L. and D.W. supervised the experimental studies. T.L. wrote the manuscript with help from N.G., D.W. and N.S. All authors read the manuscript and approved the final version.

## Conflicts of interest

The authors declare no conflict of interest.

## Supporting information


**Table S1** List of primers used in this study.

**Table S2** List of *R. solanacearum* strains used in this study.

**Table S3** Adapted chromatographic separation and MS settings.

**Table S4** Mass‐spectrometry transitions monitored for each analyte.

**Figure S1** Polymorphisms in *Brg11‐EBEs* of *R. solanacearum* host species do not affect activation by RipTALs from different *R. solanacearum* phylotypes.
**Figure S2** RipTAL‐YFP fusion proteins are localized to the nucleus and are expressed to similar levels.
**Figure S3** Non‐edited image of 5′RACE in tomato wildtype and *Δ1/2‐Brg11‐EBE* mutants.

## Data Availability

Data sharing not applicable to this article as no datasets were generated or analysed during the current study
