# Supplementary material for: An ancient cis‐element targeted by Ralstonia solanacearum TALE‐like effectors facilitates the development of a promoter trap that could confer broad‐spectrum wilt resistance
Source: Plant Biotechnol J. 2023 Oct 23;22(3):602–16. doi: 10.1111/pbi.14208 (PMC10893940; doi:10.1111/pbi.14208)
Supplement: Supplementary file 3 — Table S3 Adapted chromatographic separation and MS settings. [file PBI-22-602-s001.docx]

| Table S3: adapted chromatographic separation and LC-MS settings | | |
| --- | --- | --- |
| time | % solvent A  (water, 0.1% aq. formic acid (FA), 0.05% HFBA) | % solvent B  (acetonitrile, 0.1% FA, 0.05% HFBA) |
| 0 | 98 | 2 |
| 0.5 | 98 | 2 |
| 3.5 | 20 | 80 |
| 4.5 | 10 | 90 |
| 5.5 | 5 | 95 |
| 6 | 5 | 95 |
| 6.5 | 1 | 99 |
| 7 | 1 | 99 |
| 7.5 | 98 | 2 |
| 10 | 98 | 2 |
| Flow rate 12 µl/min. All solvents were LCMS grade. MS settings: Optiflow Turbo V ion source with SteadySpray T micro electrode (10–50 μl/min); ion spray voltage: +4800 V; nebuliser, heater gas = nitrogen, 25 and 45 psi; curtain gas, nitrogen, 30 psi; collision gas, nitrogen, medium; source temperature, 200 °C; entrance potential, ±10 V; collision cell exit potential, ±10 V; scan time 10 ms. | | |
