# Supplementary material for: An ancient cis‐element targeted by Ralstonia solanacearum TALE‐like effectors facilitates the development of a promoter trap that could confer broad‐spectrum wilt resistance
Source: Plant Biotechnol J. 2023 Oct 23;22(3):602–16. doi: 10.1111/pbi.14208 (PMC10893940; doi:10.1111/pbi.14208)
Supplement: Supplementary file 4 — Table S4 Mass‐spectrometry transitions monitored for each analyte. [file PBI-22-602-s003.docx]

| Table S4: Transitions monitored for each analyte**.** | | | | | |
| --- | --- | --- | --- | --- | --- |
| Q1 mass (Da) | Q3 mass (Da) | declustering potential (volts) | collision energy  (volts) | compound | quantifier ion |
| 89.1 | 72 | 22 | 14 | putrescine 1 | + |
| 90.1 | 73 | 22 | 14 | putrescine 2 | - |
| 146.1 | 129 | 20 | 20 | spermidine 1 | - |
| 146.1 | 72 | 20 | 20 | spermidine 2 | + |
| 203.2 | 112 | 48 | 15 | spermine 1 | - |
| 203.2 | 129.1 | 48 | 24 | spermine 2 | + |
| 131.1 | 114.1 | 22 | 20 | agmatine 1 | - |
| 131.1 | 72.1 | 22 | 20 | agmatine 2 | + |
| 136.1 | 119.1 | 22 | 20 | ^13^C_5_-agmatine 1 | - |
| 136.11 | 76.1 | 22 | 20 | ^13^C_5_-agmatine 2 | + |
| 210.1 | 192 | 60 | 22 | tryptophane D5 1 | - |
| 192.1 | 150.1 | 100 | 22 | tryptophane D5 2 | + |
