# Supplementary material for: An ancient cis‐element targeted by Ralstonia solanacearum TALE‐like effectors facilitates the development of a promoter trap that could confer broad‐spectrum wilt resistance
Source: Plant Biotechnol J. 2023 Oct 23;22(3):602–16. doi: 10.1111/pbi.14208 (PMC10893940; doi:10.1111/pbi.14208)
Supplement: Supplementary file 5 — Figure S1 Polymorphisms in Brg11‐EBEs of R. solanacearum host species do not affect activation by RipTALs from different R. solanacearum phylotypes. Figure S2 RipTAL‐YFP fusion proteins are localized to the nucleus and are expressed to similar levels. Figure S3 Non‐edited image of 5′RACE in tomato wildtype and Δ1/2‐Brg11‐EBE mutants. [file PBI-22-602-s002.pdf]

Figure S1

a

Host	gene	*Brg11-EBE*	ATG*
S.lycopsersicum	*SlADC1*	GAGGGGGTAGCCGGGGC	134
C.annuum	*CaADC1*	GAGGAGGTAGCCGAGGC	168
A.thaliana	*AtADC2*	GAGGGGGTAGCCGAGGC	191
M.trunactula	*MtADC1*	GGGGGGGTAGCCGGGGC	118
G.max	*GmADC1*	GAGGGGGAAGCCGGGGC	134

b

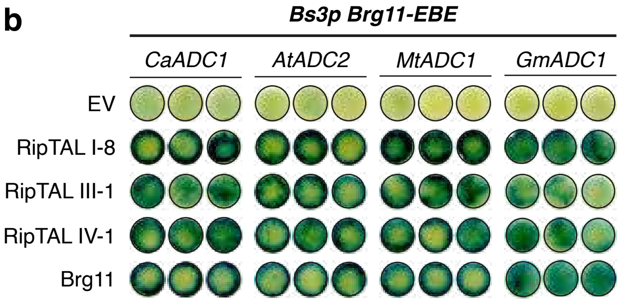

Figure S1: Polymorphisms in *Brg11-EBEs* of *R. solanacearum* host species do not affect activation by RipTALs from different *R. solanacearum* phylotypes.

(a) Nucleotide polymorphisms across *Brg11-EBEs* of different *R. solanacearum* host species. Polymorphisms are shown in red font. Distances of *Brg11-EBEs* to the ATG start codon are indicated (ATG*) (b) GUS-staining of *N. benthamiana* leaf discs defines compatibility of *Brg11-EBEs* from different *R. solanacearum* host species with RipTALs from different *R. solanacearum* phylotypes. GUS staining was conducted two dpi with *A. tumefaciens* strains containing depicted *35S* promoter-driven *RipTAL* genes (left) along with strains containing depicted promoter:*GUS* constructs (top). The GUS reporter is driven by a pepper *Bs3* minimal promoter (*Bs3p*). *Brg11-EBEs* from different *R. solanacearum* host species were inserted into the *Bs3* promoter (*Bs3p Brg11-EBE*), as shown in (a).

Figure S2

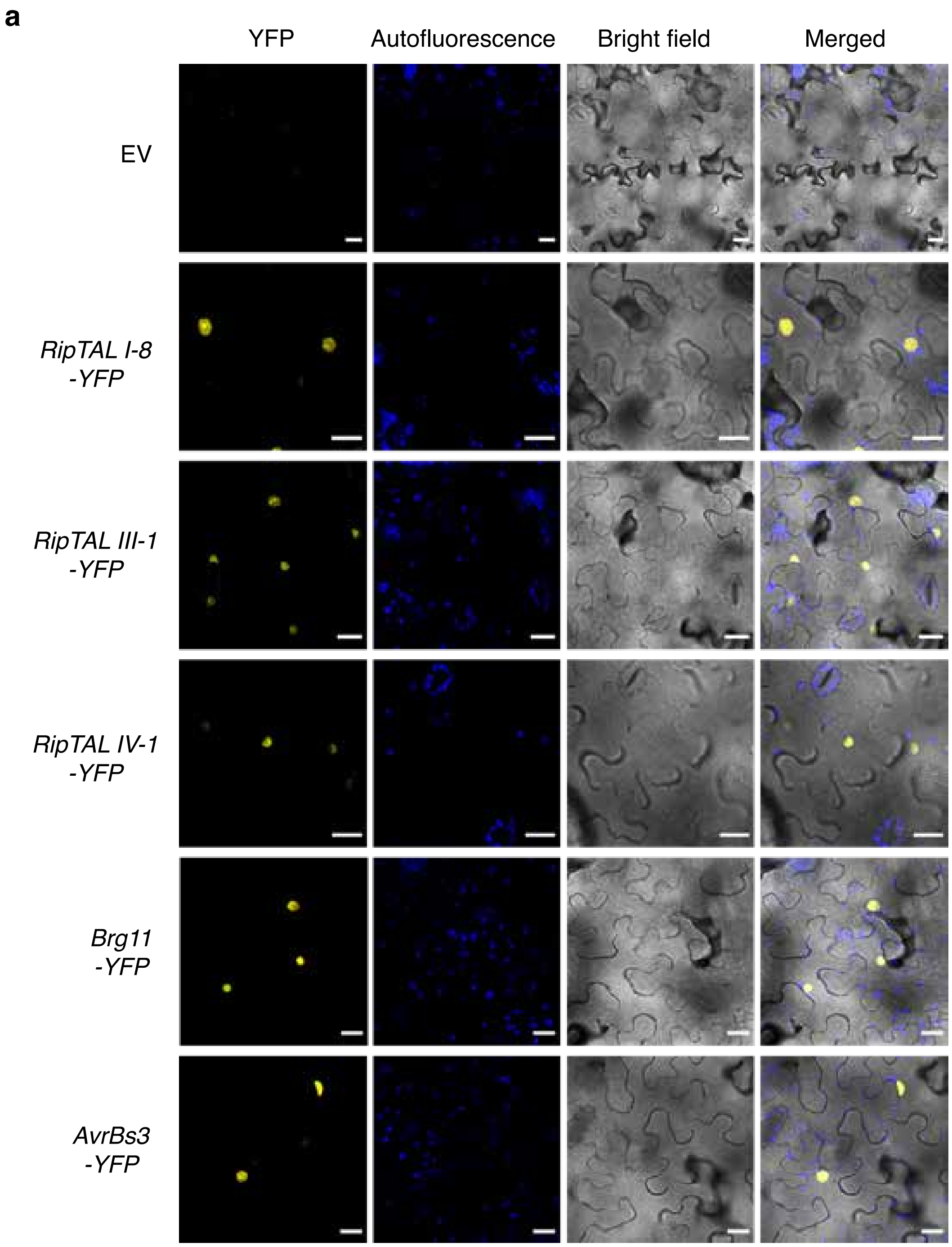

Figure S2: RipTAL-YFP fusion proteins appear to be nuclear localised and have similar levels of expression.

35S promoter-driven T-DNAs encoding the illustrated RipTAL-YFP fusion proteins were introduced into *N. benthamiana* leaves via *A. tumefaciens*-mediated delivery. Images were taken at 48 hpi using a Leica SP8 confocal microscope. Scale bars represent 33 µm.

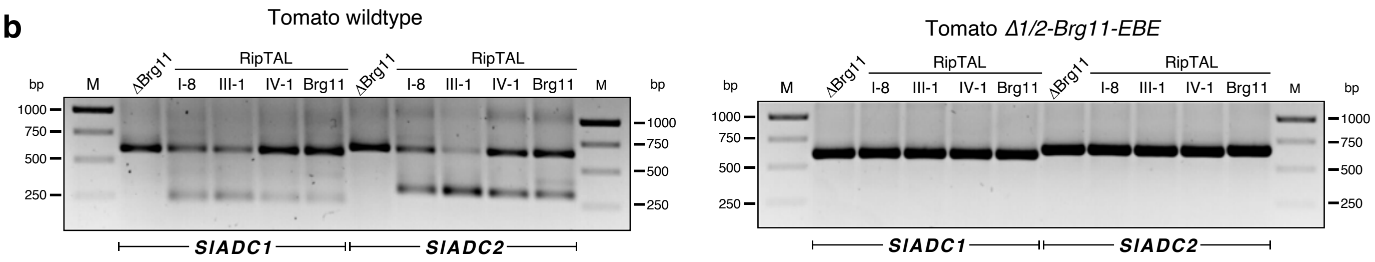

Figure S3: Unedited image of 5’RACE performed on tomato WT and *Δ1/2-Brg11-EBE* mutant plants as described in Figure 4.
